# Intensive and pharmacological care in times of COVID-19: A “special ethics” for emergency?

**DOI:** 10.1186/s12910-020-00562-7

**Published:** 2020-11-19

**Authors:** Enrico Marinelli, Francesco Paolo Busardò, Simona Zaami

**Affiliations:** 1 Department of Anatomical, Histological, Forensic and Orthopaedic Sciences, Sapienza University of Rome, Rome, Italy; 2 Department of Excellence of Biomedical Sciences and Public Health, University Politecnica delle Marche, Ancona, Italy

**Keywords:** COVID-19, Emergency, Ethics

## Abstract

**Background:**

The Authors have laid out an analysis of Italian COVID-19 confirmed data and fatality rates, pointing out how a dearth of health care resources in northern regions has resulted in hard, ethically challenging decisions in terms of granting patient access to intensive care units (ICU).

**Main text:**

Having to make such decisions certainly entails substantial difficulties, and that has led many health care professional to seek ethical guidance. The Italian Society of Anesthesia, Analgesia, Resuscitation and Intensive Care (SIAARTI) has attempted to meet that growing need by a set of recommendations, applying “clinical soundness” as a beacon standard; that approach tends to prioritize patients with higher life expectancy, which could be characterized as a “moderately utilitarian” approach. Yet, such a selection has engendered daunting ethical quandaries. The authors believe it can only be warranted and acceptable if rooted in a transparent decision-making process and verifiable, reviewed criteria. Moreover, the authors have stressed how clinical experimentation in a pandemic setting is a subtext of great interest from an ethical perspective. In Italy, no drug therapy and trials were undertaken for COVID-19 patients for a rather long period of time. When the epidemic was already circulating, an intervention proved necessary on the system of administrative procedures, aimed at expediting the authorization and validation of protocols, then bogged down by bureaucracy. A new system has since been instituted by a government decree that was signed about one month after the first Covid-19 case was officially recorded in the country. Such a swift implementation, which took just a few weeks, is noteworthy and proves that clinical trials can be initiated in a timely fashion, even with a pandemic unfolding. The concerted, action of supportive care and RCTs is the only way to attain effective forms of treatments for COVID-19 and any other future outbreak.

**Conclusions:**

The authors have arrived at the conclusion that the most effective and ethically sound response on the part of any national health care system would be to adequately reconfigure its organizational mechanisms, by making clinical trials and all related administrative procedures consistent with the current state of emergency.

## Background

Italy currently has the fourth highest number of confirmed COVID-19 cases, after Russia, Spain and the United Kingdom, coupled with the sixth highest fatality rate in Europe [1].

Possible explanations for those numbers seem to point towards three distinct factors:

- the nation’s demographic make-up (high average age, 23% of the population age 65 or older);
- the definition of COVID-19 related death, which in Italy has come to include all COVID-19 positive casualties; such a standard may have led to an overestimation of the infection as a cause of death factor, in light of the fact that over 70% of those perished have been found to have two or three comorbidities;
- The role played by the *testing strategy,* which had been initially implemented in a rather extensive fashion, and has been eventually limited to highly symptomatic individuals in need of hospitalization, as requested by national health care authorities; such a policy change may have resulted in relatively higher fatality rates, which is apparent when compared to extremely extensive testing policies put in place by other nations, such as South Korea.

Those three elements identified by Onder et al. [2] have undoubtedly played a key role, along with the disproportionate concentration of cases in Italy’s northern regions, in causing oversaturation and the ensuing shortages in intensive care unit beds during the first weeks of the epidemic. Although Italy’s health system is generally highly regarded, and can rely on 3.2 hospital beds per 1000 people (as compared with 2.8 in the United States), it has been impossible to meet the needs of so many critically ill patients simultaneously [3].

## Main text

### Ethical decision-making against the backdrop of finite health care resources

In Italy, the prevalent therapeutic approach overall has been to implement standardized respiratory and circulatory support for seriously ill patients, whereas mildly symptomatic patients have been supervised with no therapeutic intervention. Such a monotherapy-based course of action, which has left out a significant share of patients who would have needed it due to shortages in intensive care unit beds in the hardest-hit regions, has brought to the forefront the ethical quandary of having to decide which patients to prioritize on account of the limited resources available.

The difficulties and extraordinary burden of having to make such decisions have led to many doctors seeking ethical guidance. As a response to that need, the Italian Society of Anesthesia, Analgesia, Resuscitation and Intensive Care (SIAARTI) has issued a set of recommendations, grounded in a “clinical soundness” standard, which tend to lean towards prioritizing patients with higher life expectancy, which could be defined as a “moderately utilitarian” approach, in the face of not enough resources to meet everyone’s needs [4]: giving artificial ventilation to patients who are unlikely to survive anyway would in fact mean denying the same care to others with higher chances of surviving and recovering. By that rationale, some have suggested that it might be necessary to set an age limit for admission to critical care units. The utilitarian reasoning is after all deemed ethically acceptable by various groups, such as the Jewish community [5].

Such a stance is of course liable to be criticized as discriminatory against elderly and otherwise unhealthy patients, and possibly running counter to universal principles whose vital nature should never be demeaned, not even in times of pandemic. That argument has been recently reasserted by international institutions such as the UNESCO, which has released a statement on ethical issues and COVID-19, drawing attention to the need to care for those most at risk because of “poverty, discrimination, gender, illness, loss of autonomy or functionality, elder age, disability, ethnicity…”, and stressing how such groups are likely to be even more vulnerable in emergency circumstances [6].

The Italian Committee for Bioethics (CNB), in an opinion released on 8th April 2020, has drawn upon some of the principles spelled out in the UNESCO statement, and criticized any selection criteria outside of clinical standards, which are viewed as the only appropriate guidance for managing access to critical care units; the Committee has however acknowledged triage as a valuable tool to that end. Patient selection criteria ought to be grounded in the principles of preparedness, adequacy and the capability to meet current needs. If the preparedness-based strategy should turn out to be lacking in terms of ensuring the highest benefit to every patient, those for which the treatment can be more effective and with the higher chances of surviving should be prioritized; that assessment needs to take into account not only patients who are physically in the facilities, but also those examined at an earlier time and found to be in critical conditions. The CNB opinion has not been unanimously approved: a minority position has in fact criticized the vague and ill-defined nature of the “higher chances of survival” standard, arguing that in cases of identical clinical evaluations, it would be far preferable to apply the standard of “higher life expectancy”, in agreement with the SIAARTI position [7].

In the United Kingdom, NICE-issued guidelines recommend a triage phase centered around patient fragility, although they offer a somewhat vague set of indications when it comes, for instance, to the assignment of artificial ventilators [8]. Hence, in a context of insufficient resources, decisions are bound to be made at the local level, which entails the risk of creating a sort of “lottery effect”, i.e. applying different criteria based on the location and facilities in which each patient is hospitalized.

The first hand experiences of doctors in the front line all over the world show that the issue of setting ethically acceptable priorities, when operating with meager resources, cannot be sidestepped.

To deal with that issue, shared decision-making pathways have been devised, of which a primary distinctive trait is to separate health care professionals who carry out therapeutic procedures from those who operate at the triage level (thus establishing who is to be admitted to critical care units), so as to ease the psychological and ethical burden borne by medical crews; secondly, the triage phase needs to be based on clearly defined parameters, meaning that the highest possible degree of transparency must be guaranteed throughout the process of defining priority standards.

In that regard, even before the Coronavirus pandemic broke out, a standard was set by which only those with the higher chances for short-term survival were to be admitted into intensive care units, such as patients with no known comorbidities, for instance [9]. Although age is not mentioned in the framework, the age factor is bound to carry a certain weight in terms of inclusion criteria. Broadly speaking, even though it might be possible, and even advisable, to discuss and plan in advance on several aspects, in real life situations any decision is influenced by, and must conform with, the current emergency setting and the scarce resources available. All debates centered around ethics have a tendency to gloss over legal aspects; still, we believe that it is essential to take into account the fallout resulting from possible violations of legal rights of all those involved. In that respect, it would be remiss to overlook a key point: all decisions made on a case-by-case basis, thus not based on a recognized and officially acknowledged scale of priorities, are liable to be contested and impugned as unwarranted, unsound, arbitrary or even discriminatory [10].

Decisions about the allocation of finite resources are likely to be called into question as well, as it is to be expected when ethical quandaries are at play [11]. Said decisions are however as likely to be accepted as the decision-making process is transparently articulated, intelligible and accessible by all those involved (patients and their family members, operators, health care facility officials and the public opinion).

It is remarkably challenging to give the “right” answer to an ethical quandary. We are not authoritative enough to weigh in on whether the long-term survival or the life-expectancy standards ought to be applied as the more ethically sound one. We have on the other hand realized that irrespective of the prioritizing standard adopted, several institutions have illustrated the characteristics that any sound decision-making process should have, when defining the allocation of scarce resources. According to a recent statement by the University of Sidney, any suitable procedure aimed at resource allocation during the current COVID-19 pandemic should directly lay out “the reasons for those decisions, who made them, and the possibility of revising decisions in the light of new evidence or new relevant considerations” [12].

In an ideal scenario, all those who contributed to the decision should be heard and given the chance to appeal. Nevertheless, such a time-consuming process may be hard to implement when decisions must be made swiftly, and answers are immediately required in order to meet pressing needs under fast-evolving circumstances. Clarity and transparency in the decision-making process under such demanding conditions could well constitute the only viable, albeit partial, solution should professionals be accused of having violated individual rights.

### Ethical dilemmas arising from non-conventional treatment

Another relevant topic pertaining to the realm of ethics has to do with the administration of innovative treatment under emergency conditions. That point has great practical relevance as well, since the therapeutic aspect may well have contributed to determining the high Coronavirus fatality rate, particularly in Italy. The Italian path has tragically laid bare all the flaws of standardized oxygen therapy in patients who spiral into severe respiratory failure, which is often impervious to oxygen therapy. The clinical records for such patients point to elevated D-dimer levels along with fibrinogen decreases and lower platelet levels, and a deterioration of coagulation factors, which is consistent with the histopathological outcome of capillary microtrombosis. Antagonism of IL6 receptors, for instance, may inhibit the massive release of cytokines, thus preventing or delaying an evolution towards massive capillary microthrombosis. In turn, a plasma transfusion may prove helpful by providing specific antibodies and coagulation factors.

Unlike what happened in Italy, in other countries (China, France, Spain, US) a large number of patients have received off-label and compassionate use therapies such as chloroquine, idroxychloroquine, azithromycin, lopinavir-ritonavir, favipiravir, remdesivir, ribavirin, interferon, convalescent plasma, steroids, and anti–IL-6 inhibitors, based on either their in vitro antiviral or anti-inflammatory properties, immune therapy with convalescent plasma.

In China and elsewhere, from the initial stages of the epidemic, various trials have been carried out using antiviral, anti-inflammatory or anti-malarial drugs, at times in combination, and plasma of recovered COVID-19 patients [13]. The results of such trials have likely contributed to molding the general therapeutic approach towards COVID-19 patients.

As a matter of fact, in Italy no drug-based therapeutic program was undertaken in COVID-19 cases for a long time, nor were any trials carried out. Only recently has the Italian Medicines Agency (AIFA) announced that Italy is set to participate in two WHO-sanctioned phase-3 studies (“Solidarity Trial”), aimed at assessing the degree of effectiveness and safety of the drug remdesivir in hospitalized COVID-19 adult patients. Research will be conducted at the Sacco hospital in Milan, the Polyclinic Hospital of Pavia, the University Hospital of Parma, and Rome’s National Institute for Infectious Diseases “Lazzaro Spallanzani”. In addition, AIFA has authorized a study, due to be coordinated by the Pascale Institute of Naples, to evaluate the efficacy of tocilizumab, a humanized monoclonal antibody against the interleukin-6 receptor (IL-6R), mainly administered for the treatment of rheumatoid arthritis [14]. As for the immune therapy with convalescent plasma, the first Italian protocol was not released until 23rd April 2020 [15].

Kalil has recently issued a warning on the compassionate use of experimental drug therapies, arguing that “numerous drugs that have been highly promising in vitro for other infectious diseases have failed in clinical studies”, adding that many such drugs “have a variety of adverse effects, including QT prolongation, torsades de pointes, hepatitis, acute pancreatitis, neutropenia, and anaphylaxis” and “could potentially increase the risk of cardiac death”. He also pointed out that “it is critical to evaluate these drugs in studies that have a concurrent control group”, which could be constituted by “the standard of care with or without placebo”. The placebo group will always be safer (in terms of possible adverse effects) than the experimental group, because patients in the placebo group will receive the established standard of care [16].

We agree with Kalil when he argues that: “If the disease is not 100% lethal and it is not known whether the experimental drug would help or harm a patient (ie, a situation with true equipoise), then it is ethical to conduct an RCT. Without a control group, it is not possible to accurately determine the harms of any experimental drug”.

During an outbreak, the type of Randomized Controled Trials (RCTs) that should be prioritized are the ones with an adaptive design, because they are able to rapidly accept or reject multiple experimental therapies throughout the trial, while being adequately gauged for meaningful clinical outcomes. With the current COVID-19 pandemic, RCTs have been launched around the world, including an adaptive trial sponsored by the NIH [17]. In Italy, with the epidemic already running for weeks, it has proven necessary to intervene on administrative procedures in order to expedite the system of authorization and validation of protocols, which was bogged down by bureaucracy. The new, streamlined system has been phased in through a government decree that was enacted about one month after the first Covid-19 case had been recorded in the country [18]. Such systemic dysfunctions have however delayed the start of controlled experimentation of new forms of treatment, and that by itself may have contributed to the high fatality rate.

It is noteworthy for initiatives of such a broad scope to have been implemented in just a few weeks, and proves that clinical trials can be swiftly initiated even in the middle of a pandemic. The rapid and simultaneous combination of supportive care and RCTs is the only way to find effective and safe treatments for COVID-19 and any other future outbreak.

## Conclusion

The glaring discrepancy between Italy’s fatality rate and other countries’ may at least partly be explained away by a therapeutic approach largely based on refraining from any initiative, other than oxygen therapy, which could have fostered the onset of severe and extremely severe manifestations, in turn resulting in the oversaturation of critical care units, especially in the country’s northern regions. Ultimately, the high fatality rate could have been brought on by factors other than high average population age, including therapeutic choices. As for the daunting challenges arising from the need to ration scarce emergency care resources, the current scenario has driven the definition of new “professional-behavioral standards”, in the form of new recommendations and guidelines from scientific societies and health care institutions. In that regard, two prominent criteria have been outlined: higher survival rates in the short term, and life expectancy (taking into account comorbidities). Both such standards have been deemed ethically acceptable; yet each one of them is liable to be called into question and criticized, as it is generally the case when facing ethical quandaries of such a magnitude. From a merely practical standpoint, guidelines and official recommendations, specific ones for each professional setting, need to be complied with; it is nonetheless just as essential to guarantee a high degree of transparency in the decision-making process, the thorough documentation of the motives determining each and every decision, and accessibility of such elements for each party involved in the process. Any failure to prioritize such pivotal aspects may entail allegations of individual rights violations, and even result in medico-legal consequences.

In addition, that key aspect brings to the forefront emergency response and reaction capacities through the experimentation of potentially effective drugs, but not to the expense of ethical and practical values of scientific evidence.

Such considerations suggest that the degree of swiftness and effectiveness with which health care institutions manage to reconfigure their ordinary set-up, from the standpoints of logistics and health care availability, is crucial in determining the most ethically sound and fruitful way to handle emergency and disaster circumstances.

From such a perspective, the creation of a permanent system of streamlined, responsive and immediately operational Randomized Control Trials (RCTs) may prove instrumental in future epidemics to help reduce the rates of morbidity and mortality.

## Data Availability

N/A.
